# Physiological analyses of traits associated with tolerance of long-term partial submergence in rice

**DOI:** 10.1093/aobpla/plu058

**Published:** 2014-09-30

**Authors:** Yoichiro Kato, Bertrand C. Y. Collard, Endang M. Septiningsih, Abdelbagi M. Ismail

**Affiliations:** International Rice Research Institute, DAPO Box 7777, Metro Manila, Philippines

**Keywords:** Flooding stress, rainfed lowland rice, shoot elongation, stagnant flooding, tillering

## Abstract

Floods are major constraints to crop production worldwide. In low-lying, flood-prone areas of the tropics, longer-term partial submergence (stagnant flooding [SF]) greatly reduces rice yield. This study assesses shoot growth and several physiological mechanisms associated with SF tolerance in rice. Five rice genotypes with contrasting responses to SF were evaluated in field ponds. Following transplanting, floodwater was gradually increased at a rate of ∼2 cm day^−1^ to reach a final depth of 50 cm and then maintained until maturity. Although plants were not fully submerged, the yield was reduced by 47 % across genotypes compared with those grown under control conditions (6.1 vs. 3.3 t ha^−1^). This reduction was mainly attributed to the reduction in biomass caused by reduced light interception and leaf growth above the water. Stagnant flooding also reduced panicle number per unit area by 52 % because of reduced tillering. Shoot elongation rate kept pace with rising floodwater and correlated positively with leaf growth and biomass production. Conversely, stem non-structural carbohydrate (NSC) concentration correlated negatively with shoot elongation rate, suggesting that fast-elongating genotypes actively consume NSCs to avoid complete submergence. Moderate shoot elongation rate strongly and positively correlated with grain yield under SF; however, elongation at rates >2.0 cm day^−1^ was associated with reduced harvest index due to a smaller panicle size and increased lodging. Tolerant varieties were found to be either inherently tall or elongate moderately with rising floodwater. Our studies suggest that to improve tolerance of SF an appropriate phenotype should combine both of these traits. Fine-tuning for optimum shoot elongation with rising floodwater is, therefore, a priority for future work.

## Abstract (precis)

Long-term stagnant flooding (SF, 50 cm water depth) greatly reduces rice yield. We assessed physiological mechanisms associated with SF tolerance in contrasting rice genotypes. SF reduced yield by 47% due to low biomass caused by reduced light interception and leaf growth above water; and reduced panicle number by 52% because of low tillering. Shoot elongation correlated positively with leaf growth and biomass production, but negatively with stem nonstructural carbohydrates (NSC). Tolerant varieties were either inherently tall or elongate moderately (<2.0 cm d^−1^) with rising floodwater. Optimum shoot elongation with rising floodwater is therefore a priority for future breeding work.

## Introduction

Flooding seriously affects plant survival in natural ecosystems and in farmlands worldwide. Complete submergence is highly damaging to most plant species, including rice (*Oryza sativa*). More than 16 % of rice lowlands worldwide and over 20 million hectares of rainfed lowlands in Asia are adversely affected by floods every year (Singh *et al.* 2011). Rice fields in these flood-prone areas are subject to either transient flash floods leading to total submergence (hereafter referred to as submergence) or to long-term partial floods (stagnant flooding, SF), and both often occur in the same field within one cropping season (Mackill *et al.* 1996). Submergence causes complete inundation for a few days to as long as 2 weeks, while SF that is often ∼25–50 cm deep only partially submerges the shoot. It is commonly referred to as medium-deep or semi-deep flooding (hereafter called SF) and usually persists for a few weeks or months. In contrast, deepwater areas experience prolonged flooding of over 50 cm for most of the season (Mackill *et al.* 1996).

Yield loss due to floods ranges from 10 to 100 % depending on flood duration, depth and floodwater conditions (Ismail *et al.* 2013). So far, a limited number of high-yielding varieties tolerant of submergence have been developed and commercialized while even fewer high-yielding genotypes have been identified which tolerate SF (Vergara *et al.* 2014). Traditional varieties still predominate in flood-prone lowland farms and rice yield is low, ranging from 0.5 to 2.0tha^−1^, less than half that of irrigated rice (Mackill *et al.* 2012).

Quiescence or escape are two contrasting strategies that enable rice to cope with different types of floods (Jackson 2006; Colmer and Voesenek 2009; Voesenek and Bailey-Serres 2009; Colmer *et al.* 2014). To date, mechanisms bringing about quiescence or escape that involve genetic, molecular and physiological aspects have been intensively studied (Ismail *et al.* 2009, 2012, 2013; Bailey-Serres *et al.* 2010; Colmer *et al.* 2014). Submergence-tolerant, low-yielding landraces such as FR13A from eastern India have been identified as early as 1950s (Mackill *et al.* 1996; Bailey-Serres *et al.* 2010). These adopt the quiescence strategy and a major QTL controlling submergence tolerance of this genotype, named *SUB1*, has been located on chromosome 9 and cloned (Xu *et al.* 2006). *SUB1* induces quiescence by suppressing the ethylene-activated shoot elongation under submergence, reducing carbohydrate consumption and enhancing survival (Fukao *et al.* 2006; Schmitz *et al.* 2013). Recently, submergence-tolerant modern varieties (Sub1 varieties) were developed by the introgression of the *SUB1* gene into popular varieties through marker-assisted breeding (Siangliw *et al.* 2003; Septiningsih *et al.* 2009, 2013; Mackill *et al.* 2012; Collard *et al.* 2013). These Sub1 varieties can survive ∼2 weeks of complete submergence and they typically provide 1–3 t ha^−1^ yield advantage over the original varieties in flood-prone rainfed lowlands (Ismail *et al.* 2013). Efforts are ongoing at the International Rice Research Institute (IRRI) to improve submergence tolerance in rice further by identifying additional QTLs additive to *SUB1* (Septiningsih *et al.* 2012). At the other extreme, fast submergence-induced shoot elongation (escape strategy) is a characteristic of rice and wild-plant species that grow in flood-prone ecosystems (Colmer and Voesenek 2009). Escape is only of major benefit to submerged terrestrial species if it leads to emergence from the water surface. In rice, at least both the quiescence and escape strategies are initiated by the accumulation of the volatile hormone ethylene inside submerged plant tissues (Jackson 2008). However, neither strategy on its own leads to successful tolerance of SF. Fast elongation underwater gives very tall and spindly plants that suffer from severe lodging (Mackill *et al.* 1996) and subsequent mortality (Singh *et al.* 2011). On the other hand, underwater quiescence is also inappropriate because plants are not usually completely submerged. Clearly, an additional adaptive strategy is needed. This conclusion is backed-up by our recent studies showing that neither quiescence nor fast elongation growth is, by themselves, compatible with achieving high yields under SF (Vergara *et al.* 2014). While rice varieties that can respond to the slowly rising water of SF by elongating their stems or leaves (escape) are probably needed, the desirability of a quiescence component is less clear. The first generation of cultivars introgressed with *Sub1* to suppress submergence-induced elongation did not perform well under SF conditions because of their short stature. More recently, however, elite breeding lines with the *SUB1* locus were identified that are better suited to SF conditions (Collard *et al.* 2013).

The objective of the present study is to evaluate growth and physiological traits associated with SF tolerance in rice. In particular, we focus on shoot elongation responses to rising floodwater during the vegetative stage to examine whether the faster elongation typical of deepwater rice would be a benefit under SF, compared with moderately taller genotypes or the semidwarf types possessing slower elongation ability with rising water. Selection of the optimum phenotype will guide breeding strategies for tolerance to SF conditions.

## Methods

### Plant materials

The following five rice genotypes were used to assess tolerance of partial submergence. IRRI154 is a high-yielding variety developed for irrigated ecosystems in the Philippines and is sensitive to complete submergence. IRRI119 was bred for the rainfed lowlands. It carries the *SUB1* gene but is moderately tall. PSB Rc18-Sub1 (Rc18-Sub1 hereafter) and Swarna-Sub1 are near-isogenic varieties of Rc18, a popular variety in the Philippines and Swarna, a popular variety in India and Bangladesh, into which *SUB1* has recently been introgressed (Ismail *et al.* 2013). Both Rc18-Sub1 and Swarna-Sub1 are semidwarf types under well-drained conditions. IRRI154 and IRRI119 were considered to be SF-tolerant in the rice breeding programme at IRRI. Rc18-Sub1 and Swarna-Sub1 are SF-intolerant (Collard *et al.* 2013). Madhukar is a deepwater rice variety from eastern India.

### Setup of field trials

Experiments were conducted at the IRRI farm in Los Baños, Philippines (14°11′N, 121°15′E, altitude 21 m) during the dry season (January to May) and wet season (July to November) of 2012. The soil (0–20 cm) at the site is an Aquandic Epiaquoll with 25 % sand, 35 % silt, 40 % clay, pH (H_2_O) 7.4, 23.0 g total C kg^−1^, 2.0 g total N kg^−1^, 25.9 mg Bray-II P kg^−1^, 1.35 cmol exchangeable K kg^−1^, 0.38 mg exchangeable Zn kg^−1^ and a cation exchange capacity of 41.3 cmol kg^−1^. Weather data were obtained from the meteorological station at the IRRI farm: mean values of air temperature, solar radiation and total rainfall of 2012 were 24.7 °C in the dry season and 25.0 °C in the wet season, 16.6 MJ m^−2^ day^−1^ in the dry season and 14.1 MJ m^−2^ day^−1^ in the wet season, and 487 mm in the dry season and 1464 mm in the wet season, respectively.

Two adjacent fields with similar soil properties (Singh *et al.* 2011) were used for two experiments, one control and the other under SF. In the control trial, 2–3 cm water depth was maintained from transplanting until 4–5 days before harvest when the field was drained. For the SF treatment, a deepwater pond cum irrigation and drainage facility (0.15 ha) was used. Water depth was maintained at 2–3 cm from 0 to 7 days after transplanting (DAT), then increased twice a week at a rate of 1.43 cm day^−1^ during the early vegetative stage (from 7 to 21 DAT) and three times a week at a rate of 2.14 cm day^−1^ during the late vegetative stage (from 21 to 35 DAT). Then, a water depth of 50 cm was maintained from 35 DAT until maturity. This gradual increase in water depth is typical of the long-term stagnant floods in some areas of tropical Asia (Singh *et al.* 2011). The trials were laid out in a randomized complete block design with four replications in each trial. However, because of the difficulty of maintaining two different water levels in numerous sub-plots in one field, the two treatments were applied in adjacent field plots and considered as separate experiments with randomization within each. Plot size was 30 m^2^ (5 m × 6 m).

Seeds were sown in a seedbed prepared in an upland field and seedlings were irrigated daily with a sprinkler. Two 21-day-old seedlings were transplanted per hill at a hill spacing of 20 × 20 cm on 5 January and 10 July 2012 for the dry season and the wet season, respectively. Fertilizers were incorporated as basal: 35 kg P ha^−1^, 66 kg K ha^−1^ and 5 kg Zn ha^−1^ in the dry season and 26 kg P ha^−1^, 50 kg K ha^−1^ and 5 kg Zn ha^−1^ in the wet season. Nitrogen was split-applied at 80 kg ha^−1^ as basal and 40 kg ha^−1^ at 30 DAT in the dry season; 60 kg ha^−1^ as basal and 30 kg ha^−1^ at 30 DAT in the wet season. Insects, diseases and weeds were carefully controlled using approved pesticides, fungicides and herbicides.

### Measurements

The height of 20 plants in each plot during the vegetative stage (from 10 to 50 DAT) was measured weekly to determine the shoot elongation rate. Cumulative plant height was plotted against time in terms of DAT. Elongation rates were calculated as the slopes of regression lines.

In the dry season, chlorophyll values were estimated with a SPAD-502 chlorophyll meter (Minolta Co., Ltd, Japan) (Takai *et al.* 2010), stomatal conductance of the abaxial sides of the uppermost fully expanded leaf was measured for 12 plants per plot using an SC-1 leaf porometer (Decagon Devices, Inc., Pullman, WA, USA) in the morning (0900 to 1100 h) under clear sky conditions. We also determined the soil and plant analyzer development (SPAD) values of the same leaves used for stomatal conductance measurements. Leaf water potentials of IRRI154, IRRI119 and Swarna-Sub1 were measured at 50 and 51 DAT at midday (1100–1300 h) using the uppermost fully expanded leaves on three plants per plot with a pressure chamber (Soil Moisture Equipment Corp., Santa Barbara, CA, USA).

To monitor aerial canopy growth, the fraction of radiation intercepted was measured every week at six positions per plot by a linear photosynthetic active radiation ceptometer (AccuPAR, Decagon Devices, Inc., Washington, USA) at midday (1100–1300 h). The ceptometer was placed on the water surface, so the values in the SF trial reflected the interception by the canopy above the water. The amount of radiation interception was calculated by multiplying the daily fraction of radiation intercepted and incident radiation measured at the meteorological station, which is <500 m away from the trials. The cumulative above-ground dry weight (as described below) was plotted against cumulative intercepted radiation and the values of radiation-use efficiency (biomass/intercepted radiation) were calculated from the slopes of the regression lines forced through the origin.

Concentrations of non-structural carbohydrates (%NSCs) were determined weekly. One to two medium-sized plants were collected from each plot, and stems were immediately excised and frozen in liquid N_2_, and then freeze-dried. Concentrations of soluble sugar and starch were measured using procedures explained in Ismail *et al.* (2009). Samples were assayed for soluble sugars using anthrone reagent, and starch concentration was determined after hydrolysis with amyloglucosidase, followed by glucose assay using glucose oxidase (Sigma Chem. Co., MO, USA). Non-structural carbohydrate concentration was expressed as the sum of the concentrations of soluble sugars and starch.

To determine tiller number, above-ground biomass and leaf area index, 12 hills (0.48 m^2^) were sampled from each plot, seven to eight times during the vegetative growth stage. Plants were pulled from the soil in the control treatment. In the SF treatment, plants were first cut at the water surface and the remaining below water shoot bases pulled from the soil. The stem number of all plants was counted. Leaf area above water was determined by a leaf area meter (LI-3000, LI-COR, Lincoln, NE, USA) after separating the samples into green leaves and stems and expressed as leaf area index based on the area of harvested hills (0.48 m^2^). The dry weight of each component was determined after oven drying at 80 °C for 72 h.

Days to heading (80 % of panicles reaching heading) was recorded. At maturity (90 % of spikelets turning yellow), the number of surviving plants and panicles was counted in a 4.48-m^2^ area where grain yield was determined. Twelve hills were randomly chosen to measure plant height, above-ground biomass, harvest index (ratio of grain weight to above-ground biomass) and yield components. Plants were separated into straw and panicles. Panicles from all 12 hills were hand-threshed and filled spikelets were separated from unfilled spikelets by submerging them in tap water. The number of filled spikelets and unfilled spikelets was determined to calculate spikelets per panicle and filled-grain percentage. Samples were oven-dried at 80 °C for 72 h. Grain yield was determined from a 4.48-m^2^ area in each plot and adjusted to a moisture content of 0.14 g H_2_O g^−1^.

### Statistical analyses

Data were analysed using the generalized linear model procedure (SAS Institute, 2003). Individual analyses of variance were conducted separately for each trial and season based on a randomized block design to assess varietal differences. The effects of water depth and the genotype × water depth interaction were assessed by combined analysis of variance from the two separate treatment plots. Differences were compared by Fisher′s least-significant difference (LSD) tests at the 5 % probability level.

## Results

### Variety x water regime interaction on grain yield and other agronomic traits

The effects of season, water (control vs. SF), genotype × water and season × water on grain yield were statistically significant (Table 1). However, genotype × water × season was only significant for harvest index. This means that screening for SF tolerance would be effective in both wet and dry seasons.

**Table 1.** *F* values and level of significance following analysis of variance of rice yield and yield attributes. GY, grain yield; BIO, biomass; HI, harvest index; PAN, panicles m^−2^; SPN, spikelets per panicle; FG, %filled grain; GW, grain weight; d.f., degree of freedom. *, ** represent significance at *P* < 0.05 and *P* < 0.01, respectively; NS, not significant.

	d.f.	GY	BIO	HI	PAN	SPN	FG	GW
Season (*S*)	1	40**	1.4_NS_	133**	14**	2.9_NS_	8**	0.7_NS_
Water (*W*)	1	442**	278**	93**	544**	2.1_NS_	13**	48**
Genotype (*G*)	4	26**	8**	44**	26**	27**	0.7_NS_	389**
*G* × *S*	4	1.0_NS_	13**	6.8**	0.8_NS_	1.3_NS_	3.4*	13**
*S* × *W*	1	16**	11**	11**	4.7*	0.5_NS_	11**	0.1_NS_
*G* × *W*	4	22**	20**	2.9*	42**	2.8*	1.8_NS_	6.5**
*G* × *S* × *W*	4	1.4_NS_	0.9_NS_	2.7*	0.6_NS_	0.5_NS_	1.2_NS_	0.5_NS_

On average, SF reduced grain yield by 37 % in the dry season and by 47 % in the wet season, with a genotypic range of 12–83 % across seasons (Table 2). IRRI154 (high-yielding variety bred for irrigated rice paddies) had the highest yield under SF in both seasons, with the least yield reduction. On the other hand, Swarna-Sub1 showed the highest yield in the control but the lowest under SF. Although yield reduction from SF was lower in the deep water variety Madhukar than in Swarna-Sub1 or Rc18-Sub1, this landrace yielded by far the least grain, under control conditions than the other more modern varieties.

**Table 2.** Grain yield (g m^−2^) of five rice genotypes under the control (C) and stagnant flood (SF) in the dry and wet seasons of 2012. Different letters following mean values indicate statistical significance (*P* < 0.05).

	2012 dry season			2012 wet season		
	C	SF	SF/C (%)	C	SF	SF/C (%)
IRRI154	628^b^	554^a^	88	636^b^	400^a^	63
IRRI119	670^b^	444^b^	66	663^ab^	333^ab^	50
Madhukar	469^c^	325^c^	69	416^d^	249^bc^	60
Rc18-Sub1	631^b^	311^c^	49	559^c^	183^cd^	33
Swarna-Sub1	739^a^	348^c^	47	707^a^	119^d^	17
Mean	627	397	63	596	257	43

Stagnant flooding stress significantly reduced survival (Table 3), although the effects were more severe on Rc18-Sub1 and Swarna-Sub1. They showed lower plant survival under SF stress compared with the other three genotypes. Generally, yield reduction under SF stress was mainly associated with reduction in shoot biomass (37 %), while harvest index was less reduced (17 %). IRRI154 ranked second in biomass production under SF after Madhukar, while Swarna-Sub1 had the lowest biomass under SF. Harvest index was the lowest in Madhukar as a consequence of severe lodging while the scale of reduction in harvest index caused by SF was also the greatest in this deepwater rice (by 33 %) and in Swarna-Sub1. Flowering date was delayed under SF by 7 days, but varietal differences in the delay were not related to genotype performance.

**Table 3.** Plant biomass, harvest index, days to heading (DTH) and survival at maturity of five rice genotypes grown under control (C) and SF. Data are mean values of dry and wet seasons in 2012. Different letters following mean values within each column indicate statistical significance (*P* < 0.05). ^†^From transplanting.

	Biomass (g m^−2^)			Harvest index			DTH (days)^†^		Survival (%)	
	C	SF	SF/C (%)	C	SF	SF/C (%)	C	SF	C	SF
IRRI154	1316^b^	1065^ab^	81	0.41^a^	0.39^a^	93	69^d^	78^c^	100^NS^	90^ab^
IRRI119	1452^ab^	964^b^	66	0.40^ab^	0.34^b^	86	73^c^	76^c^	100	83^b^
Madhukar	1294^b^	1102^a^	85	0.31^c^	0.24^d^	77	68^d^	68^d^	100	93^a^
Rc18-Sub1	1358^b^	662^c^	49	0.38^b^	0.31^c^	82	76^b^	83^b^	100	74^c^
Swarna-Sub1	1493^a^	586^c^	39	0.42^a^	0.32^bc^	77	83^a^	99^a^	100	62^d^
Mean	1383	876	63	0.38	0.32	83	74	81	100	80

Among the yield components, panicles per square metre was most associated with grain yield under SF across the two seasons (*r* = 0.92**, *n* = 10), with an average reduction of 52 % (Table 4). Reduction in panicles per square metre under SF stress was least in Madhukar (24 %) and greatest in Swarna-Sub1 (77 %). On an average, SF effects on other components were <10 %. The difference in spikelets per panicle between the control and SF was not significant, although it was reduced by SF in Madhukar. Filled-grain percentage was significantly higher under SF than the control because of the compensatory effect of reduced spikelets per square metre. Grain weight was slightly reduced by SF stress.

**Table 4.** Yield components of five rice genotypes under control (C) and SF. Data are means of two trials conducted during the wet and dry seasons of 2012. Different letters following mean values within each column indicate statistical significance (*P* < 0.05).

	Panicles (no. m^−2^)			Spikelets (no. panicle^−1^)			Filled grains (%)			Grain weight (mg)		
	C	SF	SF/C (%)	C	SF	SF/C (%)	C	SF	SF/C (%)	C	SF	SF/C (%)
IRRI154	282^b^	183^a^	65	118^b^	124^b^	105	70_NS_	82_NS_	118	24.1^c^	21.9^d^	91
IRRI119	218^c^	126^b^	58	119^b^	120^bc^	101	76	79	104	29.5^a^	27.9^a^	94
Madhukar	165^d^	125^b^	76	112^b^	103^c^	92	74	75	101	27.9^b^	26.1^b^	94
Rc18-Sub1	281^b^	100^c^	36	106^b^	107^c^	101	74	83	112	23.5^c^	23.1^c^	98
Swarna-Sub1	323^a^	75^d^	23	139^a^	161^a^	116	74	78	106	19.1^d^	19.2^e^	101
Mean	254	122	48	119	123	104	74	79	108	24.8	23.6	95

### Growth and biomass accumulation

**Figure 1.**
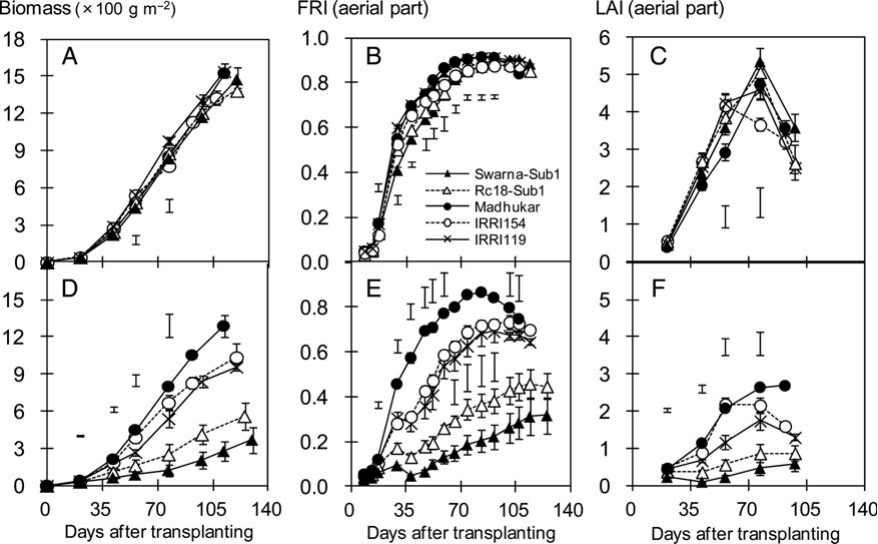
Time course of shoot biomass, fraction of radiation intercepted (FRI) and leaf area index (LAI) in the control (A–C) and under SF (D–F) during the wet season of 2012 (mean ± SE). Bars indicate LSD at *P* < 0.05.

**Figure 2.**
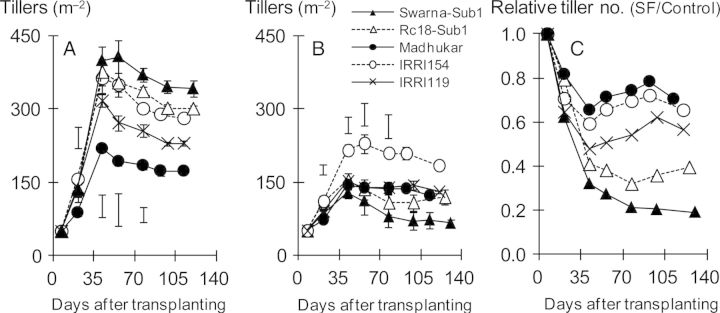
Time course of tiller number in the control (A) and under SF (B), and the ratio of tiller number under SF relative to the control (C) during the wet season of 2012 (mean ± SE). Scale bars indicate LSD at *P* < 0.05.

When plant biomass was assessed on the basis of radiation intercepted and radiation-use efficiency, it became apparent that light interception above water appears to be the main factor limiting biomass production under SF stress (Table 5). Cumulative radiation intercepted closely, correlated positively with biomass at maturity in the dry season (*r* = 0.95**, *n* = 5) and in the wet season (*r* = 0.99**, *n* = 5). IRRI154 and Madhukar intercepted the largest amount of radiation energy under SF, while Swarna-Sub1 intercepted only 37 % of incident radiation compared with the control. On the other hand, radiation-use efficiency was little changed by SF stress. Furthermore, varietal differences in plant biomass under SF correlated positively with the fraction of radiation intercepted by the aerial canopy (at 49 DAT, *r* = 0.97**, *n* = 5) and leaf area index above water (at 49 DAT, *r* = 0.99**, *n* = 5; Fig. 1). Under SF, Madhukar had the largest canopy at the vegetative stage, while Swarna-Sub1 had the smallest canopy. Varietal differences in tiller number under SF also corresponded well with differences in light interception and leaf area (Fig. 2). Varietal differences in tiller number under SF stress were not related to those in the control (Fig. 2A and B). Swarna-Sub1 produced the highest number of tillers per square metre in the control but the lowest number under SF. The relative tiller number (SF/Control) ranged from 75 % of controls for Madhukar to 20 % for Swarna-Sub1 (Fig. 2C). Tiller number under SF also correlated positively with the fraction of intercepted radiation (at 56 DAT, *r* = 0.972**, *n* = 5) and plant biomass (at 56 DAT, *r* = 0.999**, *n* = 5) under SF.

**Table 5.** Cumulative radiation intercepted and radiation-use efficiency for five genotypes under control (C) and SF. Data are means of the wet and dry seasons of 2012. Different letters following mean values within each column indicate statistical significance (*P* < 0.05).

	Cumulative radiation intercepted (MJ)			Radiation-use efficiency (g MJ^−1^)		
	C	SF	SF/C (%)	C	SF	SF/C (%)
IRRI154	951^c^	778^ab^	82	1.43_NS_	1.47_NS_	103
IRRI119	1052^b^	743^b^	71	1.44	1.40	97
Madhukar	965^c^	829^a^	86	1.39	1.40	101
Rc18-Sub1	1030^bc^	515^c^	50	1.39	1.34	96
Swarna-Sub1	1146^a^	425^d^	37	1.39	1.33	96
Mean	1029	658	64	1.41	1.39	98

**Figure 3.**
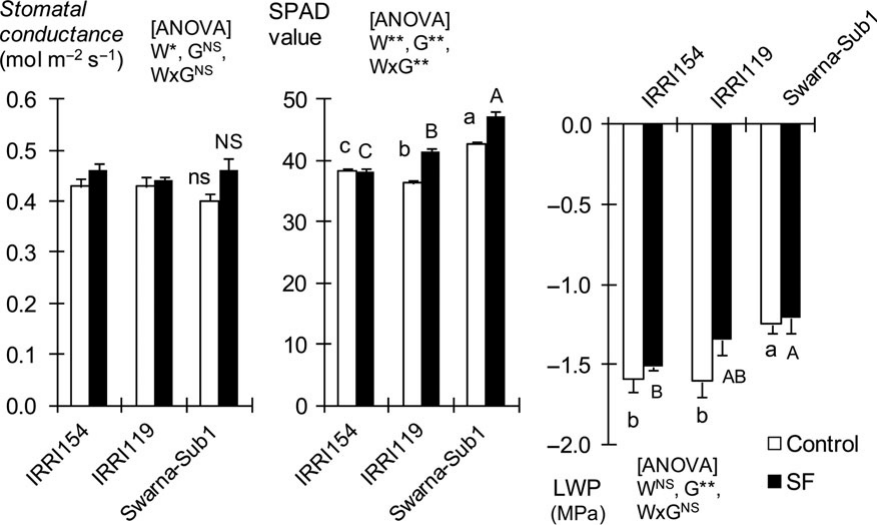
Stomatal conductance (*g*_s_), SPAD value and leaf water potential (LWP) at 50 and 51 DAT during the dry season of 2012 (mean ± SE). Different letters indicate statistical difference (*P* < 0.05). SF, stagnant flooding; G, genotype; W, water regime; *W* × *G*, water × genotype interaction.

The relatively smaller change in radiation-use efficiency caused by SF was also reflected in the observation related to aspects of leaf photosynthetic capacity (Fig. 3). The SPAD chlorophyll value, midday leaf water potential and stomatal conductance were all slightly higher under SF than in the control, even for Swarna-Sub1, though some of the variation was statistically insignificant.

### Shoot elongation and dynamics of NSCs

**Figure 4.**
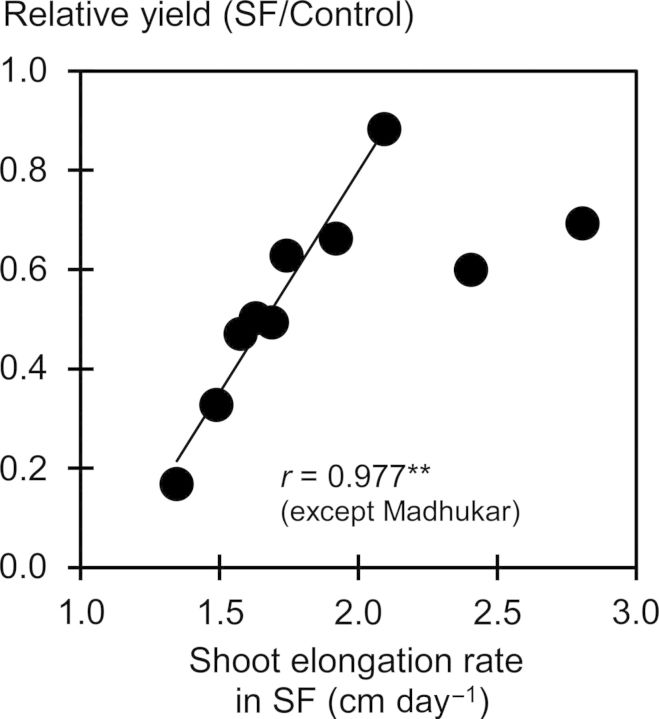
Relationship between shoot elongation rate (SER) under SF and relative rice yield (ratio of yield under SF to that in the control).

Plants were 9 cm taller at maturity in the wet season than in the dry season across water regimes (Table 6). However, under SF, shoot elongation rate at the vegetative stage was slower in the wet season than in the dry season. Under SF, shoot elongation was fastest in Madhukar, followed by IRRI154, and slowest in Swarna-Sub1. Varietal differences in shoot elongation corresponded well with those in light interception (at 49 DAT, *r* = 0.96**, *n* = 5) and leaf area (at 49 DAT, *r* = 0.96**, *n* = 5) under SF, but not in controls. As a consequence, shoot elongation rates of <2.0 cm day^−1^ closely correlated with relative yield (ratio of yield in SF to that in controls; *r* = 0.98**, *n* = 8; Fig. 4). However, elongation at rates greater than this value were associated with reduced harvest index caused by severe lodging and reduced panicle size, as experienced with the deepwater genotype Madhukar.

**Table 6.** Plant height at maturity and shoot elongation rate (SER) at the vegetative stage for five genotypes under control (C) and SF. Data are means of the dry and wet seasons of 2012. Means followed by different letters within each column indicate statistical significance (*P* < 0.05).

	Plant height (cm)			SER (cm day^−1^)		
	C	SF	SF/C (%)	C	SF	SF/C (%)
IRRI154	111^c^	140^b^	126	1.34^c^	1.92^b^	143
IRRI119	134^b^	140^b^	104	1.65^b^	1.78^c^	108
Madhukar	157^a^	177^a^	113	1.94^a^	2.61^a^	135
Rc18-Sub1	113^c^	131^c^	116	1.34^c^	1.59^d^	119
Swarna-Sub1	101^d^	122^d^	121	1.11^d^	1.46^e^	132
Dry season	117	139	119	1.55	2.02	130
Wet season	129	145	112	1.40	1.72	123

Varietal differences in elongation in response to SF stress were observed. In IRRI 119, shoots elongated little in response to rising water (108 % of the control) but in IRRI154 elongation in SF was much faster (143 % of control) even though both lines are considered tolerant of SF (Vergara *et al.* 2014). To characterize this response in each genotype further, shoot elongation under SF stress was divided into constitutive (inherent) and adaptive (facultative) elongation. Madhukar was inherently tall and showed higher SF-induced elongation. IRRI119 had intermediate stature with slow flooding-induced (facultative) elongation, while IRRI154 is semidwarf but with higher facultative elongation. Rc18-Sub1 and Swarna-Sub1 are both semidwarf with the latter showing a relatively higher facultative elongation rate.

**Figure 5.**
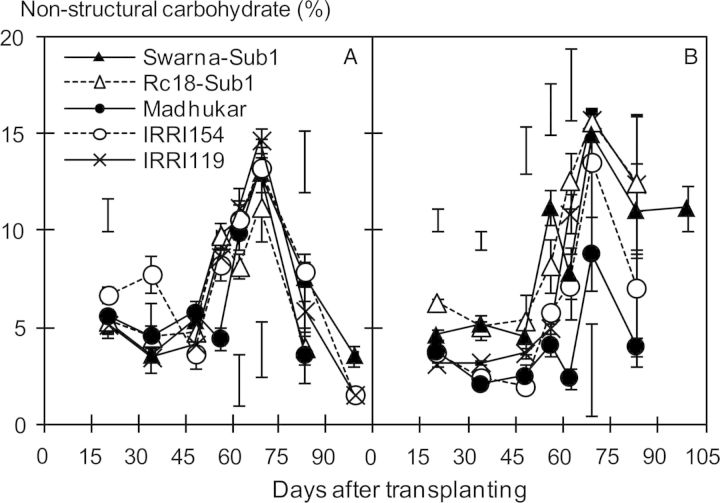
Time course of concentration of stem NSC (%, dry weight basis) in the control (A) and SF (B) trials during the dry season of 2012 (mean ± SE). Scale bars indicate LSD at *P* < 0.05.

**Figure 6.**
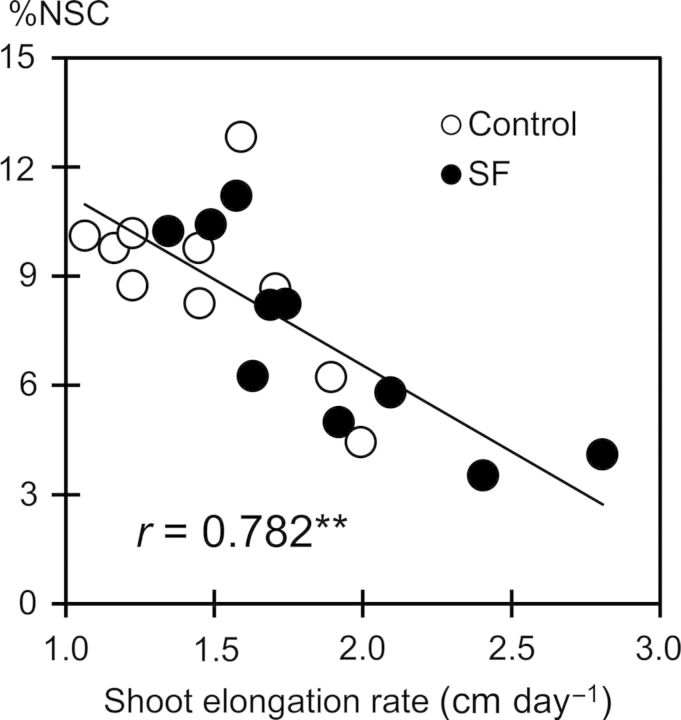
Relationship between SER during the vegetative stage and concentration of stem NSC (%NSC) at 56 DAT in the dry and wet seasons of 2012. SF, stagnant flooding.

The dynamics of NSCs in stems under SF were different from those in the control (Fig. 5). The concentration of NSC was relatively lower under SF for the first 2 months with increasing water depth before sharply increasing during the reproductive stage (after 60 DAT) to reach values higher than controls. However, %NSC in Madhukar given SF remained below control levels, possibly due to its faster elongation. In contrast to the plant biomass results, %NSC under SF stress was lower in Madhukar but highest in Rc18-Sub1 and Swarna-Sub1 during the vegetative stage. Non-structural carbohydrate concentration was correlated negatively with shoot elongation rate at the end of the vegetative stage (*r* = 0.78**, *n* = 20; Fig. 6).

## Discussion

Our results demonstrate that regulation of shoot elongation has a major role in SF tolerance. As has been widely recognized, control of shoot elongation is important for submergence tolerance both during germination and early establishment as well as during vegetative stage (Ismail *et al.* 2012). However, the desirable elongation response to flooding is dependent on the flood type and stage of development. The present study clarified aspects of the connection between elongation and adaptation to SF that have not been commented on previously, e.g. in Colmer and Voesenek (2009). First, we observed that leaf photoassimilation capacity is much less affected by stress from SF compared with the impact on leaf area. This was true even for SF-intolerant genotypes. The reduction of radiation-use efficiency, which is often used as an indicator of the balance between canopy photosynthesis and respiration (Sinclair and Muchow 1999), was reduced by <5 % under SF (Table 5). Leaf water potential or stomatal conductance was also less affected by SF stress, indicating that the balance between water uptake by the root, plant hydraulic conductivity and water vapour loss from stomata was not impaired (Fig. 3). To date, we do not have direct evidence to confirm whether the internal longitudinal diffusive resistance to gases, especially to oxygen moving to the roots, is affected by SF. Accordingly, it will be useful, in future work, to examine changes in internal root, stem and leaf anatomy, including the formation of aerenchyma (the snorkel-like conduits for gas diffusion; Kordan 1974) in response to rising floodwater and their contribution to leaf photosynthesis, plant respiration and root aeration as possible flood-avoidance traits.

SPAD chlorophyll values were higher under SF than in the controls (Fig. 3), although we observed faster degradation in the lower leaves that were submerged (Y. Kato, IRRI, Philippines, pers. observ.). This indicates that degradation of chlorophyll in leaves above the water surface is not evident under SF. This contrasts with the situation during flash flooding when the entire canopy is inundated. Here, not only is NSC depleted during submergence but leaf chlorophyll is considerably decayed due to oxidative stress upon de-submergence. Both responses affect plant survival detrimentally (Ella *et al.* 2003*a*; Das *et al.* 2005). Degradation of leaf chlorophyll under flash floods is triggered by ethylene accumulation, which the *SUB1* gene can effectively prevent (Jackson *et al.* 1987; Ella *et al.* 2003*b*). The effects of SF stress on photoinhibition and on the generation and deactivation of reactive oxygen species in leaves at different positions on stems await further investigation.

Of the yield components we examined, panicles per square meter was the most affected by SF stress (decreased by 52 %; Table 4), agreeing with our previous studies (Singh *et al.* 2011). The average reduction in plant survival under SF was 20 % (Table 3), whereas panicles per surviving plant were reduced by 35 %. Both effects contributed to the lower number of panicles per unit area. Dynamic changes in relative tiller number (Fig. 2) suggest that varietal differences in tillering ability and biomass production under SF are largely attributable to reduced light interception and concurrent lowering of photoassimilation. However, tillering was inhibited by SF, even in Madhukar where light interception and biomass accumulation during the vegetative stage were similar under SF and control conditions (Fig. 1). These observations suggest that the suppression of tiller growth under SF happens irrespective of the availability of NSC or concurrent assimilation at the onset of rising floodwater and that these tiller buds would not recover at later stages. This is supported by our ancillary pot experiment (Y. Kato, IRRI, Philippines, unpubl. data) and previous study on tillering dynamics under SF (Sugai *et al.* 1999). Since reduction in tiller number directly affects rice yield under SF, anatomical, biochemical and molecular studies on this temporal tiller suppression should be considered in future studies.

It is well established that the quiescence (slow shoot elongation) strategy is especially advantageous in flash-flood prone areas where the whole shoot is submerged at a depth too great for vigorous shoot elongation to return leaves to the air, or the flooding duration is short (less than 2 weeks). On the other hand, the escape strategy with its characteristic fast underwater elongation is crucial for survival in deepwater rice areas where floodwater deepens rapidly in the field (Colmer and Voesenek 2009; Colmer *et al.* 2014). In the present study, we found that relying on either strategy alone would not be adaptive for SF. Faster shoot elongation contributed to establishing a larger aerial leaf area and higher light interception, biomass production and plant survival (Fig. 1; Table 3) compared with slower elongating plants. Associated penalties were lower %NSC, severe lodging and reduced harvest index. Although Madhukar produced a high biomass under SF, the demand for NSC to support fast elongation seemingly did not match the supply (Figs 5 and 6). Young panicles compete with stems for NSC during reproductive development (Fujita and Yoshida 1984), suggesting that Madhukar and similar deepwater varieties could potentially suffer from carbohydrate shortage for panicle and grain formation.

Our study also suggested two patterns of shoot elongation that are potentially adaptive to SF stress in modern rice varieties (Table 6). One involves genotypes with intermediate height, such as IRRI119, which are inherently tall and with little additional elongation capacity in response to rising floodwater. Rice genotypes with plant heights of 130–140 cm under non-flooded conditions would lodge badly if they elongated to even greater height when under SF. The other pattern is seen in the inherently semidwarf genotypes, such as IRRI154, where a higher elongation response with rising floodwater holds adaptive value. During the vegetative stage, the elongation of IRRI154 under SF occurred mostly in leaf sheaths and blades and not in the internodes (data not shown). Any of the semidwarf rice varieties that are suitable for SF conditions can, therefore, be expected to elongate relatively quickly and maintain sufficient leaf area above the water surface to generate adequate biomass, while avoiding excessive height that would cause lodging. Vergara *et al.* (2014) reported that elongation rate that maintains ∼50 % of the shoot height above the water surface is likely to be optimum for higher yields under SF. Relying on the quiescence strategy in taller genotypes would in contrast increase the probability of complete submergence when flooding depth exceeds the inherent height. But, similarly in semidwarf lines there is an enhanced risk of complete submergence at the seedling stage compared with genotypes with intermediate height. Further studies are likely to establish whether one or the other or a combination of the two strategies will likely result in the development of rice varieties that are more resilient in SF-affected areas.

**Figure 7.**
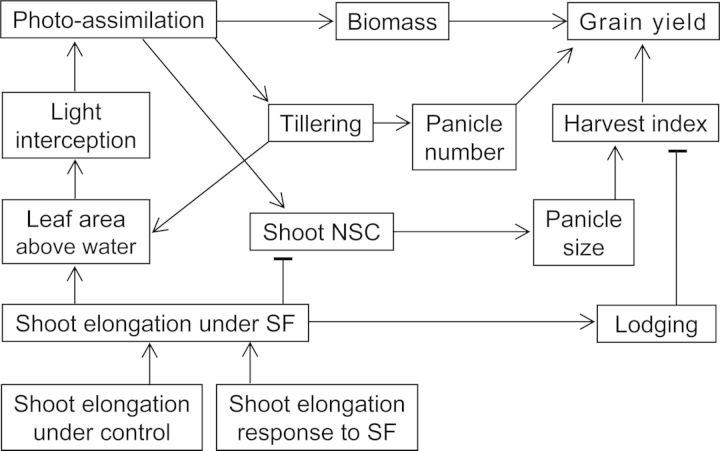
Schematic illustration of the growth and physiological traits associated with SF tolerance in rice. NSC, non-structural carbohydrates.

To summarize the mechanisms associated with SF tolerance, we propose a flowchart of shoot growth processes likely affected by SF based on this study (Fig. 7). Inherent stature and elongation response to rising floodwater determine shoot elongation rate and optimum height under SF stress. Shoot elongation is closely related to enhanced leaf area and light interception as determinants of photoassimilation under SF stress. Radiation-use efficiency is less important for photoassimilation as it is much less reduced by SF stress than is total light interception. Tillering is not related to stem NSC status but more to leaf area under SF, indicating that deficiency in concurrent assimilation due to reduced leaf area could reduce tiller formation and/or promote senescence under SF. Non-structural carbohydrate concentration in plant tissue is a result of the balance between the supply of and the demand for assimilates, but this study suggests that shoot elongation rate is the major determinant of the remaining NSCs in stems under SF stress. This is in agreement with previous studies on submergence in rice (Setter and Laureles 1996; Das *et al.* 2005). Carbohydrate depletion could reduce panicle size due to poor growth of young panicles. High shoot elongation rate is important for biomass production under SF, but it also increases the risk of lodging, thereby reducing harvest index. Accordingly, a shoot elongation rate of <2.0 cm day^−1^ strongly correlated with relative yield (SF/control; *r* = 0.98**, *n* = 8). Evidently, fine-tuning of optimum shoot elongation with rising floodwater is important for developing new rice varieties with SF tolerance. The results presented in this study could assist breeders to screen or select breeding material with tolerance to SF for targeted breeding for flood-prone environments.

## Sources of Funding

This study was supported, in part, by several donors. The authors acknowledge the support of the Bill & Melinda Gates Foundation, the German Federal Ministry for Economic Cooperation and Development (BMZ) and the International Fund for Agricultural Development (IFAD).

## Contributions by the Authors

Y.K., A.M.I., B.C.Y.C. and E.M.S. conceived the study; Y.K. and A.M.I. designed the experiments. Y.K. carried out the experiment under the supervision of A.M.I. and Y.K. and A.M.I. wrote the manuscript. All authors checked and approved the final draft.

## Conflicts of Interest Statement

None declared.
